# Editorial: Science in Mechanical Circulatory Support

**DOI:** 10.3389/fcvm.2021.676595

**Published:** 2021-05-17

**Authors:** Kiyotake Ishikawa, Bart Meyns, Navin K. Kapur

**Affiliations:** ^1^Cardiovascular Research Institute, Icahn School of Medicine at Mount Sinai, New York, NY, United States; ^2^Department of Cardiac Surgery, Universitair Ziekenhuis Leuven Gasthuisberg University Hospital, Leuven, Belgium; ^3^The Cardiovascular Center, Tufts Medical Center, Boston, MA, United States

**Keywords:** LV unloading, LV assist device, hemodynamic, experimental, clinical

The technological advances in mechanical circulatory support (MCS) devices over the last two decades have been staggering. The development of percutaneous MCS devices has allowed for the rapid and minimally invasive endovascular insertion of these lifesaving technologies. Recent data indicate that these devices not only improve systemic circulation but also offer benefits through cardiac unloading. The physiological and molecular effects of cardiac unloading confer beneficial effects on the heart and improve patient outcomes (1–3), but our knowledge in this area requires further improvement. To take maximum advantage of cardiac unloading, we must understand the device-specific and disease-dependent impact of MCS devices on cardiovascular physiology, myocardial biology, and clinical outcomes.

In this special issue Research Topic collection, eight manuscripts covering the spectrum of clinical and basic research related to MCS were collected. These studies provide new insights into the physiological impact of MCS devices (Nix et al., Kariya et al., Tschöpe et al.), and identify clinically useful prognostic markers of MCS patients (Schäfer et al. Whitehead et al., Davila et al.), and develop methods and algorithms to better manage patients with MCS (Kakino et al., Tschöpe et al.).

In the domain of basic research, Nix et al. compare the hemodynamic impact of veno-arterial extracorporeal membrane oxygenation (VA-ECMO) and Impella CP using a porcine model of acute myocardial infarction. The study reports time-dependent changes in cardiac physiology using the two devices. Although the authors found a similar reduction in myocardial oxygen consumption, its mechanism seems to act differently. A similar infarct size in the two groups confirms the importance of reducing myocardial oxygen consumption in acute MI settings for infarct size reduction. Kariya et al. study the impact of Impella support on coronary physiology. They create a new porcine model of coronary arterial dissection and study coronary flow and pressure before and after Impella support. The study demonstrates that Impella is effective in reducing cardiac work in a heart with coronary dissection, but there is a rare case that Impella might deteriorate coronary flow when there is a large intimal flap. The authors propose the expected mechanism of flow deterioration by Impella support. Kakino et al. uniquely combine an *in silico* approach with a large animal experiment. They bring in the concept of circulatory equilibrium in predicating the hemodynamic impact of MCS. After developing the framework, they validate this framework in a dog model of coronary ligation and demonstrate reliable prediction of cardiac output under MCS. The manuscript provides a comprehensive description of a framework that could be useful in the clinical management of MCS patients.

In the clinical realm, Schäfer et al. study the characteristics of patients who were treated by Impella for acute myocardial infarction-related cardiogenic shock (AMI-CS). They report lower mortality in their population compared to predicted mortality based on previous studies. Additional insight is provided for the most effective timing to start hemodynamic support in such a population. Whitehead et al., Davila et al. examine the role of right-side heart pressures in predicting the clinical outcome of AMI-CS patients treated with MCS. Whitehead et al. focus on the central venous pressure and examine its role in clinical outcomes in 132 Impella supported patients from 28 centers. Meanwhile, Davila et al. focus on right atrial pressure in 76 AMI-CS patients with MSC. Davila et al. also examine longitudinal right atrial pressure variation to determine the role of decongestion time course in predicting clinical outcomes. These studies explore the importance of right heart failure in acute MCS, which has not been well-studied. Tschöpe et al. report on how to manage a difficult case of significant hemolysis in a patient with myocarditis, supported by a combination of ECMO and Impella. Tschöpe et al. also describe an algorithm to safely wean patients from Impella based on their extensive experience. They propose a TIDE algorithm which stands for: (1) transthoracic echocardiography under full Impella-unloading; (2) impella rate reduction in single 8 to 24 h-steps according to patients hemodynamics; (3) dobutamine stress-echocardiography; and (4) right heart catheterization at rest and during exercise-testing via handgrip. Since we currently have little information on the best weaning method after acute MCS, the study provides vital information for clinicians involved in acute MCS.

Multifaceted research *in silico, in vivo*, and in humans has provided new knowledge and insights into the emerging field of acute cardiac unloading using MCS devices. We thank the authors who contributed to this special issue and hope these new findings improve patient outcomes for millions suffering from cardiovascular disease.

## Author Contributions

KI drafted the editorial and all authors made critical edits. All authors approved the final version of the editorial.

## Conflict of Interest

KI and BM serves as a PI of a research grant to the institution from Abiomed Inc. NK receives consulting/speaker honoraria and institutional grant support from: Abbott Laboratories, Abiomed Inc., Boston Scientific, Medtronic, LivaNova, MDStart, and Precardia.
